# Dual Targeting of EGFR with PLK1 Exerts Therapeutic Synergism in Taxane-Resistant Lung Adenocarcinoma by Suppressing ABC Transporters

**DOI:** 10.3390/cancers13174413

**Published:** 2021-09-01

**Authors:** Sol-Bi Shin, Dae-Hoon Kim, Da-Eun Kim, Mark Borris D. Aldonza, Yoosik Kim, Hyungshin Yim

**Affiliations:** 1 Department of Pharmacy, Institute of Pharmaceutical Science and Technology, College of Pharmacy, Hanyang University, Ansan 15588, Korea; solbi@hanyang.ac.kr (S.-B.S.); kdhsh100@hanyang.ac.kr (D.-H.K.); daeunyh@hanyang.ac.kr (D.-E.K.); 2 Department of Chemical and Biomolecular Engineering, Korea Advanced Institute of Science and Technology (KAIST), Daejeon 34141, Korea; borris@alumni.kaist.ac.kr (M.B.D.A.); ysyoosik@kaist.ac.kr (Y.K.)

**Keywords:** chemoresistance, PLK1, EGFR, ABC transporter, lung adenocarcinoma

## Abstract

**Simple Summary:**

Our previous studies led us to hypothesize that downregulation of PLK1 expression or its activity can overcome the hurdles of taxane resistance by downregulating ABC transporters. Targeting PLK1 with shRNA or non-functional mutants downregulated *ABCB1*, *ABCC9*, and *ABCG2* in paclitaxel-resistant lung adenocarcinoma (LUAD^TXR^), similar to the downregulation effects from treatment with PLK1 inhibitors. Since *EGFR* is highly expressed in LUAD^TXR^ cells, gefitinib was combined with PLK1 inhibitors. Under these conditions, LUAD^TXR^ cells tend to undergo apoptosis more effectively than parental cells, showing a synergistic effect on downregulation of ABC transporters through c-Myc or AP-1. Clinical data provide evidence for the relationship between survival rates and expressions of *PLK1* and *EGFR* in LUAD patients. Taken together, our data suggest that a combination of gefitinib and PLK1 inhibitors exerts strong synergism in LUAD^TXR^, providing a benefit to overcome the limitations associated with taxanes.

**Abstract:**

To overcome the limitations of chemoresistance, combination therapies using druggable targets have been investigated. Our previous studies led us to hypothesize that the downregulation of PLK1 expression or activity can be one strategy to overcome the hurdles of taxane resistance by the downregulation of ABC transporters. To explore this, various versions of PLK1 including a constitutively active version, kinase-dead form, and polo-box domain mutant were expressed in paclitaxel-resistant lung adenocarcinoma (LUAD^TXR^). Targeting PLK1 using shRNA or non-functional mutants downregulated *ABCB1*, *ABCC9*, and *ABCG2* in LUAD^TXR^ cells, which was similar to the downregulation effects from treatment with PLK1 inhibitors. The high expression of *EGFR* in LUAD led us to administer gefitinib, showing a markedly reduced *EGFR* level in LUAD^TXR^ cells. When gefitinib and PLK1 inhibitors were combined, LUAD^TXR^ cells tended to undergo apoptosis more effectively than parental cells, showing a synergistic effect on the downregulation of ABC transporters through c-Myc and AP-1. Clinical data provide evidence for the relevance between survival rates and expressions of *PLK1* and *EGFR* in LUAD patients. Based on these results, we suggest that a combination of gefitinib and PLK1 inhibitors exerts strong synergism in LUAD^TXR^, which helps to overcome the limitations associated with taxanes.

## 1. Introduction

Lung adenocarcinoma (LUAD) is a major subtype of non-small cell lung cancer (NSCLC), an aggressive cancer [1,2,3]. Because LUAD is rather insensitive to chemotherapy compared to the lung squamous cell carcinoma (LUSQ) of NSCLC, several lines for chemotherapy have been developed based on molecular biomarkers [4]. For treatment of non-LUSQ patients including those with LUAD, genetic mutation of EGFR is an important factor to determine the first line of chemotherapy. In second- or third-line treatment of non-LUSQ patients, taxanes and platinum are recommended [4]. The taxanes paclitaxel and docetaxel are widely used for treatment of several solid malignant tumors, and a combination of taxanes with other chemotherapeutic medicines is standard therapy for cancer patients including those with NSCLC [5,6]. Paclitaxel induces cytotoxicity through interference with the physiological function of microtubules and mitotic progression in cancer [5]. During paclitaxel-induced mitotic catastrophe, formation of reactive oxygen species was induced [5]. Although taxanes such as paclitaxel are used broadly for clinical applications, resistance against taxanes can develop in patients, which has been raised as a clinical obstacle. 

As a main cause of paclitaxel resistance, expression of ABC transporters has been proposed [7,8,9]. In taxane-resistant cancer, *ABCB1* (encoding multidrug resistance protein 1; MDR1 or P-glycoprotein) and *ABCG2* (encoding breast cancer resistance protein; BCRP) ABC transporters have been studied well as transporters of paclitaxel in several carcinomas [7,8,10]. ABC transporters have been targeted to overcome chemoresistance using specific inhibitors such as valspodar [11,12,13], dofequidar [14], tariquidar [15], and zosuquidar [16]. However, their clinical trials were discontinued due to toxicities or lack of beneficial effects [11,12,13,15,16]. Because of these difficulties with ABC transporter inhibitors, other strategies are needed to overcome the hurdles of chemoresistance in cancer.

Our recent studies revealed that paclitaxel-resistant prostate and lung cancer upregulate the level of PLK1 with ABC transporters [17]. Due to inhibition of PLK1 activity with specific inhibitors such as BI2536, volasertib, and genistein in prostate cancer, the levels of ABC transporters such as *ABCB1* and *ABCC1* were downregulated [17]. Moreover, PLK1 is a valuable target for cancer treatment, since its overexpression is correlated inversely with the survival rates of patients in several carcinomas [18]. In addition, its involvement in chemoresistance has been studied [19,20,21]. Previous studies led us to hypothesize that downregulation of PLK1 expression or its activity can be a strategy to overcome the hurdles of chemoresistance by downregulating ABC transporters. To explore this, various versions of PLK1 including a constitutively active version, a kinase-dead form, and a polo-box domain mutant were expressed in paclitaxel-resistant LUAD (LUAD^TXR^), and their effects on the expression levels of ABC transporters were observed. Since EGFR is highly activated in LUAD, combination effects between PLK1 inhibitors and gefitinib were observed in LUAD^TXR^. The current study reveals that a combination of gefitinib and PLK1 inhibitors provides a benefit to overcome the hurdles associated with taxanes-resistance in LUAD.

## 2. Materials and Methods

### 2.1. Materials

Roswell Park Memorial Institute (RPMI)-1640, fetal bovine serum, penicillin, and streptomycin were purchased from Corning Cellgro (Manassas, VA, USA). Volasertib was purchased from Selleck Chemicals (Houston, TX, USA). Genistein, paclitaxel, gefitinib, and all other chemical reagents were purchased from Sigma-Aldrich (St. Louis, MO, USA).

### 2.2. Cell Culture and Establishment of Paclitaxel-Resistant Cancer Cells

Human lung adenocarcinoma NCI-H460 and A549 cells were purchased from the Korean Cell Line Bank (KCLB, Seoul, Korea) and were authenticated before being frozen by KCLB using STR. NCI-H460 cells were cultured at 37 °C in a 5% CO_2_ humidified atmosphere in RPMI-1640 medium supplemented with 10% (*v/v*) heat-inactivated fetal bovine serum, 100 units/mL penicillin, and 100 µg/mL streptomycin. NCI-H460 and A549 cells were exposed to stepwise escalating levels of paclitaxel to produce paclitaxel-resistant cells. The concentration of paclitaxel increased 2-fold at each step of resistance from 1 nM up to 20 nM for NCI-H460^TXR^. The resistant cells were established after NCI-H460^TXR^ exposure of 30 weeks to paclitaxel. A549^TXR^ cells were developed by exposing A549 cells (Prof. Sang Kook Lee, Seoul National University, Seoul, Korea) to up to 200 nM paclitaxel over 12 months. In the NCI-H460^TXR^ cells, various versions of PLK1 mutants were expressed for additional weeks using lentiviral pLVX-TRE3G-eRFP-PLK1 and pLVX-Tet3G vectors (Clontech #631351; Palo Alto, CA, USA) in an expression system as described previously [22]. For lentiviral PLK1 shRNA, the targeting sequences of human PLK1 (gene access no. NM_005030) are AGATTGTGCCTAAGTCTCTGC at positions 245–265 (#1) and AGATCACCCTCCTTAAATATT at positions 1424–1444 (#2) as described previously [22,23].

### 2.3. Cell Viability Assay

Cell viability was measured using 3-(4,5-dimethylthiazolyl-2)-2,5-diphenyltetrazolium bromide (Sigma-Aldrich; St. Louis, MO, USA) according to the manufacturer’s protocol and a previous report [24]. Briefly, 0.8 × 10^4^ cells in each well of a 96 well-plate were treated with paclitaxel, volasertib, genistein, or gefitinb for 48 h at the indicated concentrations. Cells were treated with 5 mg/mL 3-(4,5-dimethylthiazolyl-2)-2,5-diphenyltetra-zolium bromide and incubated 37 °C for 4 h. The formazan dye was then measured with microplate reader (SpectraMax M4, Molecular Devices; Sunnyvale, CA, USA) at 540 nm absorbance. 

### 2.4. Quantitative Reverse Transcription Polymerase Chain Reaction (qRT-PCR)

Total RNA was extracted at 48 h after exposure to volasertib, genistein, or gefitinib and quantified by a NanoDrop spectrophotometer (Thermo Scientific; Wilmington, DE, USA). Next, cDNA was created with a First Strand cDNA Synthesis Kit (Thermo Scientific; Wilmington, DE, USA). After the synthesized cDNA was mixed with SYBR Green Master Mix (Bio-Rad; Hercules, CA, USA) and various sets of gene-specific primers, qRT-PCR was performed using a CFX96 Real-Time PCR system (Bio-Rad; Hercules, CA, USA). The primer sequences used are shown in Supplementary Table S1.

### 2.5. Fluorometric Caspase 3 Assay

Using the fluorogenic Ac-DEVD-AMC substrate (BD Biosciences; San Jose, CA, USA), cell lysates were incubated with substrate in reaction buffer (20 mM HEPES, pH 7.5, 2 mM DTT, and 10% glycerol) at 37 °C for 2 h. Caspase 3 activity was monitored by fluorescence emission at 430 nm (excitation at 360 nm) using microplate reader (SpectraMax M4, Molecular Devices; Sunnyvale, CA, USA). 

### 2.6. Statistical Analysis

All data are given as means ± standard errors of the means or standard deviation from at least three independent experiments. Results were analyzed for statistically significant differences using Student’s *t*-test or a two-way ANOVA test. *P*-value less than 0.05 was considered statistically significant.

### 2.7. Bioinformatics Analysis

Kaplan–Meier survival analyses and log-rank tests were used to evaluate the statistical significance of survival differences between the two groups. Heat maps were generated using the GENE-E software (Broad Institute, Cambridge, MA, USA). GSEA was performed using the Broad Institute platform (http://www.broadinstitute.org/gsea/index.jsp (accessed on 18 December 2013). Patient samples were selected and grouped for high or low mRNA expression of the selected gene or gene set. The mean value was calculated over the whole data set.

## 3. Results

### 3.1. ABC Transporters Were Regulated by Active PLK1 in Paclitaxel-Resistant LUAD

Paclitaxel, a microtubule-stabilizing agent, leads to mitotic arrest and upregulation of mitotic kinase PLK1 by interfering with cell division in cancer cells [17,25,26]. Previously, we found that the levels of PLK1 and ABC transporters were highly upregulated in paclitaxel-resistant (TXR) lung cancer cells compared with those of parental cells [17,27]. 

**Figure 1:**
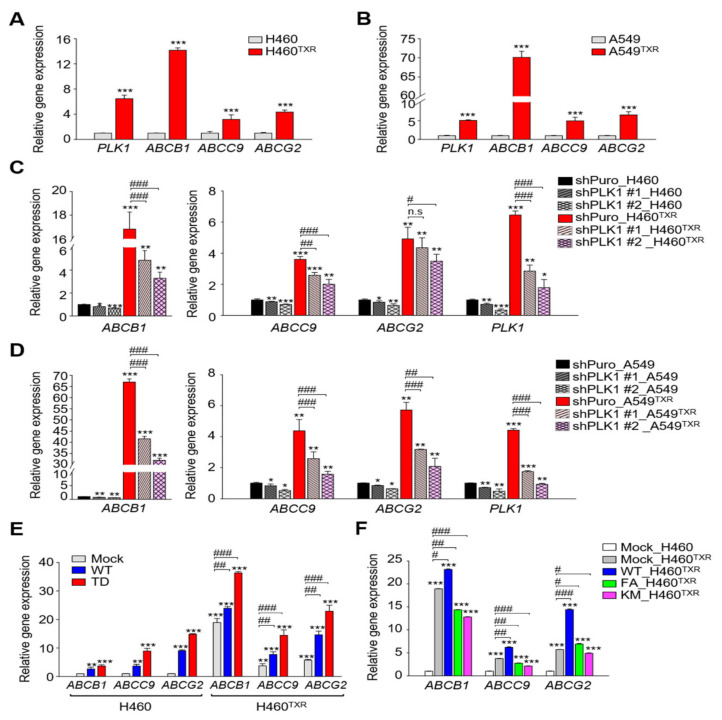
Expression levels of ABC transporters were regulated by PLK1 activity in paclitaxel-resistant lung cancer cells. (**A**,**B**) Quantitative RT-PCR was performed to evaluate the mRNA levels of *PLK1*, *ABCB1*, *ABCC9*, or *ABCG2* in parental and paclitaxel-resistant NCI-H460 (H460^TXR^) or A549 (A549^TXR^) cells. Three independent experiments were performed. The relative expression levels of mRNA were plotted. ***, *p* < 0.001. (**C**,**D**) Paclitaxel-resistant H460^TXR^ or A549^TXR^ cells were infected by lentiviral shRNA targeting PLK1 and then selected with puromycin for 48 h. Quantitative RT-PCR was performed to evaluate the mRNA levels of *PLK1, ABCB1, ABCC9*, or *ABCG2,* and the relative expression levels were plotted. Three independent experiments were performed. *, *p* < 0.05; **, *p* < 0.01, ***, *p* < 0.001 compared with shPuro of parental cells. #, *p* < 0.05; ##, *p* < 0.01; ###, *p* < 0.001 compared with shPuro of TXR cells. (**E**,**F**) ERFP-tagged vector (Mock), wild-type (WT), or catalytically active T210D (TD) of PLK1 were expressed in parental and paclitaxel-resistant NCI-H460 cells (**F**) ERFP-tagged vector (Mock), wild-type (WT) variants of PLK1 were expressed in H460^TXR^ cells. Quantitative RT-PCR was performed to evaluate the mRNA level of *ABCB1, ABCC9,* or *ABCG2*, and the relative expression of mRNA was plotted. Three independent experiments were performed. **, *p* < 0.01; ***, *p* < 0.001 compared with mock of parental H460 cells. #, *p* < 0.05; ##, *p* < 0.01; ###, *p* < 0.001 compared with compared with mock of H460^TXR^ cells.

As shown in Figure 1, the levels of *PLK1* and ABC transporters including *ABCB1* were upregulated highly in A549^TXR^ cells as well as NCI-H460^TXR^ cells (Figure 1A,B). Since PLK1 inhibitors such as volasertib and genistein enhance the therapeutic sensitivity of paclitaxel-resistant cancer [17,27], we hypothesized that blocking PLK1 would suppress chemoresistance through the downregulation of ABC transporters. To examine this, PLK1 expression was depleted using two PLK1 shRNA with different target sequences showing efficient downregulation of PLK1 [23] to observe whether ABC transporters can be downregulated in paclitaxel-resistant lung adenocarcinoma cells (Figure 1C,D). As shown in Figure 1C, the level of *ABCB1* was reduced by approximately 0.3- to 0.2-fold compared to those of the control in PLK1-depleted NCI-H460^TXR^. In PLK1-depleted A549^TXR^ cells, the level of *ABCB1* were reduced under the half-time compared to those of the control (Figure 1D, left panel). In contrast, the levels of *ABCC9* and *ABCG2* decreased compared to those of the control cells (shPuro) in PLK1-depleted NCI-H460^TXR^ and A549^TXR^ cells (Figure 1C,D, right panel). The results indicate that downregulation of PLK1 decreased the levels of ABC transporters including *ABCB1*, a main causal factor of paclitaxel resistance. 

To investigate whether the kinase activity of PLK1 affects the expression of ABC transporters, wild-type and active forms of PLK1 (T210D; TD) were expressed in NCI- H460^TXR^ cells using a doxycycline-inducible lentiviral expression system (Figure 1E). Then, qRT-PCR was performed to measure the mRNA levels of ABC transporters in NCI- H460^TXR^ cells expressing active PLK1 (T210D; TD) and wild-type PLK1 (Figure 1E). The results showed that *ABCB1* levels were highly upregulated in cells expressing active PLK1 (TD) compared with those of the mock control. *ABCB1* mRNA markedly increased by approximately 36-fold in NCI-H460^TXR^ cells expressing active PLK1 (TD) compared with the mock control of parental NCI-H460 cells, indicating that expression of *ABCB1* was regulated by active PLK1. In contrast, the levels of *ABCC9* and *ABCG2* were upregulated in NCI-H460^TXR^ cells expressing active or wild-type PLK1. The data indicate that active PLK1 can upregulate the expression of *ABCB1*, a driver of widespread chemoresistance. Next, to determine whether the activity of PLK1 is important in regulating the expression of ABC transporters, a kinase-defective mutant (K82M; KM) and a polo-box domain mutant (FA) of PLK1 were expressed in NCI-H460^TXR^ cells (Figure 1F). Quantitative RT-PCR results showed that the expression of KM and FA reduced the expression levels of *ABCB1*, *ABCC9,* and *ABCG2* by approximately half compared with those of the wild type. Therefore, expression of ABC transporters can be regulated by PLK1 activity and expression.

### 3.2. Paclitaxel-Resistant NCI-H460^TXR^ Lung Cancer Cells Underwent Apoptosis More Easily by Expression of Non-Functional PLK1

**Figure 2:**
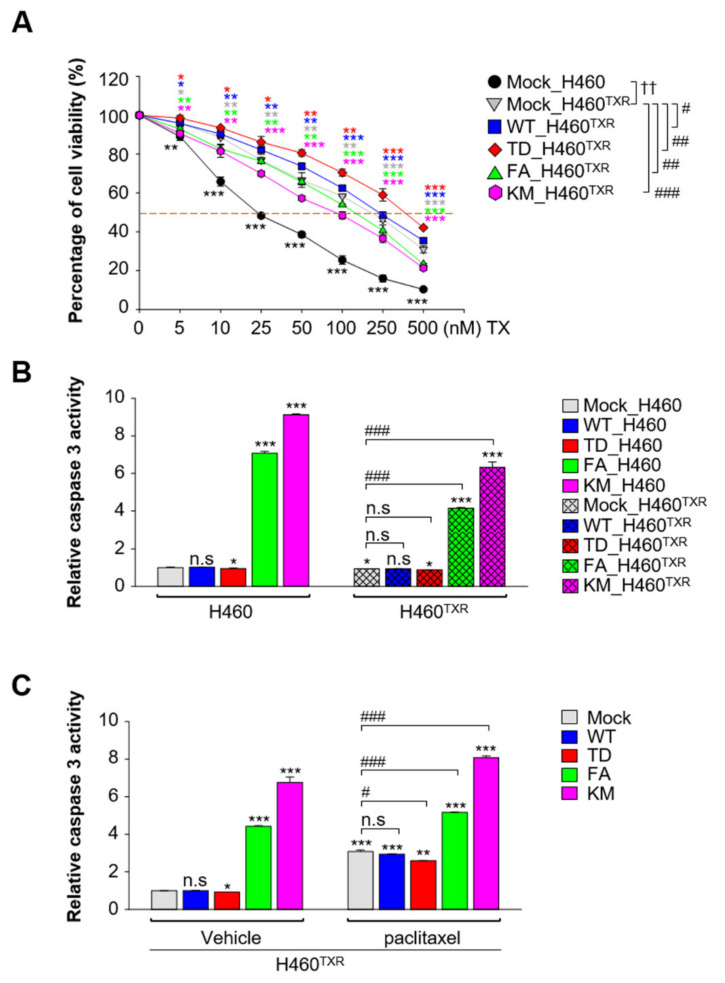
Paclitaxel-resistant NCI-H460^TXR^ lung cancer cells underwent apoptosis more easily with expression of non-functional PLK1. ERFP-tagged vector (Mock), wild-type (WT), polo-box mutant (FA), or kinase-dead K82M (KM) variants of PLK1 were expressed in parental or paclitaxel-resistant NCI-H460 (H460^TXR^) cells. Cells were selected with puromycin. (**A**) NCI-H460 and NCI-H460^TXR^ cells were grown for 48 h in the presence of 5, 10, 25, 50, 100, 250, or 500 nM paclitaxel. The percentages of viable cells were measured by cell viability assay. Three independent experiments were performed. *, *p* < 0.05; **, *p* < 0.01; ***, *p* < 0.001 compared with control (vehicle-treated). #, *p* < 0.05; ##, *p* < 0.01; ###, *p* < 0.001 compared with mock of H460^TXR^ cells. ††, *p* < 0.01 compared with mock of H460 cells. Statistically significant differences were analyzed using Student’s *t*-test or two-way ANOVA test. (**B**) Cell lysates were subjected to a fluorometric caspase 3 activity assay. The means ± SEMs (error bars) of data from at least three experiments are shown. *, *p* < 0.05; ***, *p* < 0.001 compared with mock of H460 cells. ###, *p* < 0.001 compared with mock of H460^TXR^ cells. (**C**) NCI-H460^TXR^ cells were treated with paclitaxel at 40 nM (the GI_30_ value) for 48 h. Cell lysates were subjected to a fluorometric caspase 3 activity assay. The means ± SEMs (error bars) of data from at least three experiments are shown. *, *p* < 0.05; **, *p* < 0.01; ***, *p* < 0.001 compared with vehicle-treated mock cells. #, *p* < 0.05; ###, *p* < 0.001 compared with paclitaxel–treated mock cells. n.s., not significant.

Since the expression of ABC transporters as upregulated by active PLK1 (Figure 1), sensitivity to paclitaxel was analyzed in NCI-H460^TXR^ cells expressing the catalytic active and inactive forms of PLK1 to determine whether catalytic inactive PLK1 can downregulate chemoresistance. Cell viability assays using paclitaxel were performed in NCI-H460^TXR^ cells expressing WT, TD, FA, and KM of PLK1 (Figure 2A, Supplementary Figure S1). The cell viability was around 14-fold higher in NCI-H460^TXR^ cells expressing the mock control than that of the parental cells (GI_50_ value: 18.1 nM in NCI-H460 vs. 215.4 nM in NCI-H460^TXR^). The cell viability was upregulated by expression of active PLK1 (TD), showing a GI_50_ value of 344.5 nM (vs. 215.4 nM in the mock control). Cells expressing the catalytically active form of PLK1 showed the highest cell viability, indicating that the presence of active PLK1 reduced the sensitivity to paclitaxel (Figure 2A; Supplementary Figure S1). In contrast, cell viability was downregulated by approximately half with expression of kinase-defective PLK1 (KM), showing a GI_50_ value of 97.2 nM compared with that of the mock control in paclitaxel-resistant NCI- H460^TXR^ cells (Figure 2A; Supplementary Figure S1). 

To determine whether non-functional PLK1 induces apoptosis, caspase-3 assays were performed using fluorogenic caspase 3 substrate in parental NCI-H460 and paclitaxel-resistant NCI-H460^TXR^ cells (Figure 2B). As shown in Figure 2B, caspase 3 activity was not detected in either NCI-H460 or NCI-H460^TXR^ cells expressing mock, wild-type, or catalytically active PLK1 (Figure 2B). However, expression of non-functional PLK1 in the ATP-binding domain (KM) or polo-box domain (FA) increased the caspase 3 activity compared with that of the mock in NCI-H460. This caspase activity was upregulated more in the parental NCI-H460 cells than in NCI-H460^TXR^ cells, indicating that non-functional PLK1 induces apoptosis more easily in parental NCI-H460 (Figure 2B). In addition, the caspase 3 activity of NCI-H460^TXR^ cells in the presence of paclitaxel was upregulated by expression of FA or KM of PLK1, promoting apoptosis compared with cells expressing wild-type PLK1 (Figure 2C). Therefore, the presence of kinase activity of PLK1 increased cell viability.

### 3.3. Paclitaxel-Resistant LUAD Upregulates EGFR Expression, Which Is Suppressed by Treatment with Gefitinib and PLK1 Inhibitors

**Figure 3:**
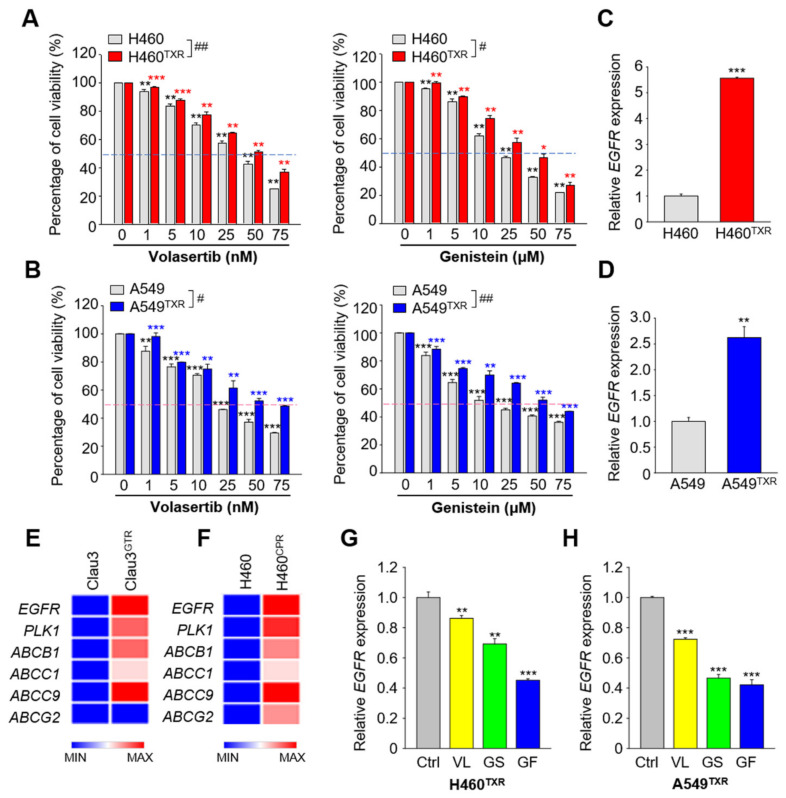
Paclitaxel-resistant LUAD was sensitive to treatment with EGFR-specific inhibitor gefitinib. (**A**,**B**) NCI-H460 (H460) and NCI-H460^TXR^ (H460^TXR^) and (B) A549 and A549^TXR^ cells were grown for 48 h in the presence of 1, 5, 10, 25, 50, or 75 nM volasertib or 1, 5, 10, 25, 50, or 75 µM genistein for 48 h. The percentages of viable cells were measured by cell viability assay. The means ± SEMs (error bars) of data from at least three experiments are shown *, *p* < 0.05; **, *p* < 0.01; ***, *p* < 0.001 compared with control of each cells. #, *p* < 0.05; ##, *p* < 0.01 compared with mock of parental H460 or A549 cells. (**C**,**D**) Quantitative RT-PCR was performed to evaluate the mRNA level of *EGFR* in (**C**) H460 and H460^TXR^ and (**D**) A549 and A549^TXR^ cells. Three independent experiments were performed. The relative expression of mRNA was plotted. ***, *p* < 0.001. (**E**) A heatmap was generated from Morpheus, showing the expression levels of *EGFR, PLK1, ABCB1, ABCC1, ABCC9*, and *ABCG2* in gemcitabine-resistant Clau3 (Clau3^GTR^) cells from gene set GSE6914. (**F**) A heatmap was generated from Morpheus, showing the expression levels of *EGFR, PLK1, ABCB1, ABCC1, ABCC9*, and *ABCG2* in carboplatin-resistant NCI-H460^CPR^ (H460^CPR^) cells based on quantitative RT-PCR. (**G**) NCI-H460^TXR^ and (**H**) A549^TXR^ cells were grown for 48 h in the presence of 80.9 nM volasertib, 55.4 µM genistein, or 18.9 µM gefitinib. The concentrations were the GI_60_ of each reagent in NCI-H460^TXR^ cells. Three independent experiments were performed. The relative expression levels of mRNA were plotted. *, *p* < 0.05; **, *p* < 0.01; ***, *p* < 0.001 compared with control.

Because NCI-H460^TXR^ and A549^TXR^ cells highly expressed *PLK1* (Figure 1), and catalytic active PLK1 expression increased cell viability (Figure 2), the PLK1-specific inhibitors volasertib and genistein were administered to NCI-H460^TXR^ and A549^TXR^ cells in a concentration-dependent manner (Figure 3). Cell viability assays showed that treatment with volasertib or genistein markedly reduced the cell viability in paclitaxel-resistant NCI-H460^TXR^ and parental NCI-H460 cells. Paclitaxel treatment showed a resistant index (RI) of 11.9 in resistant cells vs. parental cells (GI_50_: 215.4 vs. 18.1, Supplementary Figure S1). Compared with this, volasertib and genistein treatment markedly reduced the RI values to 1.7 (51.4 vs. 30.2) and 1.6 (37.8 vs. 23.2), respectively (Figure 3A; Supplementary Figure S2A). These patterns were similar in A549^TXR^ cells treated with volasertib and genistein (Figure 3B; Supplementary Figure S2B), indicating that PLK1-specific inhibitors revert paclitaxel-resistant LUAD back to sensitivity. 

As a molecular marker of LUAD, the expression levels of *EGFR* and its mutation are targetable indices to determine first-line treatment of LUAD [4]. Although we found genistein as a PLK1 inhibitor, it is also reported as an EGFR inhibitor [28] even though its inhibitory activity against PLK1 is much stronger than that against EGFR [24]. Since the RI value of genistein is better than that of volasertib, we sought to investigate the effects of EGFR in paclitaxel-resistant lung cancer. For this, the expression of *EGFR* mRNA was observed in parental and paclitaxel-resistant NCI-H460^TXR^ and A549^TXR^ cells (Figure 3C,D). The expression of *EGFR* was over 5 times or 2.5 times higher in NCI-H460^TXR^ or A549^TXR^ cells, respectively, than that of the control (Figure 3C,D). To understand the expression of *PLK1* and *EGFR* in chemoresistant lung cancer, we analyzed a published transcriptome of gemcitabine-resistant Calu3 lung adenocarcinoma cells (GSE 6914) [29]. In this analysis, the mRNA levels of *EGFR, PLK1*, and *ABCB1* were upregulated in gemcitabine-resistant Calu3 (Figure 3E). In addition, carboplatin-resistant NCI-H460 cells showed higher levels of *EGFR, PLK1*, and ABC transporters related to chemoresistance than those of parental cells (Figure 3F). Since the level of *EGFR* was high in chemoresistant lung cancer, the EGFR-specific inhibitor gefitinib was administered to NCI-H460^TXR^ and A549^TXR^ cells to determine the sensitivity to gefitinib in paclitaxel-resistant lung cancer (Supplementary Figure S2C,D). Of note, the GI_50_ values of gefitinib in NCI-H460 and NCI-H460^TXR^ cells were 16.68 µM and 12.06 µM, respectively (Supplementary Figure S2C, right panel; Table 1). NCI-H460^TXR^ cells were more sensitive to gefitinib than were the parental cells. The GI_50_ values of gefitinib in A549 and A549^TXR^ cells were 19.91 µM and 43.17 µM, respectively (Supplementary Figure S2D, right panel; Table 2). 

Next, to observe the effects of PLK1 and EGFR inhibitors on *EGFR* mRNA expression in LUAD^TXR^, qRT-PCR was performed in NCI-H460^TXR^ and A549^TXR^ cells treated with volasertib, genistein, and gefitinib at the value of GI_60_ (Figure 3G,H). The expression of *EGFR* was reduced markedly by treatment with gefitinib. Treatment with volasertib and genistein reduced *EGFR* expression by approximately 14% and 31%, respectively, in NCI-H460^TXR^ cells. In A549^TXR^ cells, volasertib and genistein treatment reduced the *EGFR* expression by approximately 28% and 53%, respectively. In contrast, administration of gefitinib strongly downregulated the expression of *EGFR* to approximately 0.4-fold that of the control in both cell types (Figure 3G,H), indicating that taxane resistance-induced *EGFR* expression is reduced effectively by gefitinib.

### 3.4. Gefitinib Treatment Results in More Effective Apoptotic Cell Death in Paclitaxel-Resistant Lung Cancer Cells Expressing High Levels of ABC Transporters Than in Non-Resistant Lung Cancer

**Figure 4:**
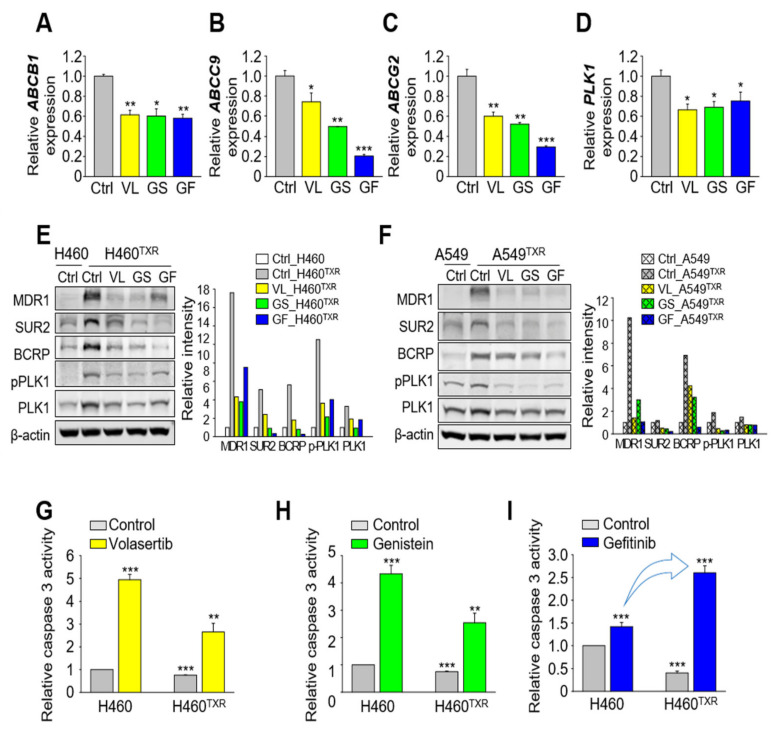
Gefitinib induces apoptosis more effectively in paclitaxel-resistant lung cancer cells expressing high mRNA levels of *EGFR*, *PLK1*, and *ABCB1* than in non-resistant lung cancer. (**A**–**F**) NCI-H460^TXR^ (**A**–**E**) or A549^TXR^ (**F**) cells were grown for 48 h in the presence of volasertib, genistein, or gefitinib at the concentration of GI_60_ of each reagent in NCI-H460^TXR^ cells. Quantitative RT-PCR was performed to evaluate the mRNA levels of (**A**) *ABCB1*, (**B**) *ABCC9*, (**C**) *ABCG2*, and (**D**) *PLK1* in parental (H460) and paclitaxel-resistant NCI-H460 (H460^TXR^) cells. Three independent experiments were performed. The relative expression levels of mRNA were plotted. *, *p* < 0.05; **, *p* < 0.01; ***, *p* < 0.001 compared with control. (**E**,**F**) Cell lysates were subjected to immunoblotting with anti-MDR1 (ABCB1), anti-SUR2 (ABCC9), anti-BCRP (ABCG2), anti-PLK1, and anti-β−actin antibodies. The relative intensities were quantified using LI-COR Odyssey software (Li-COR Biosciences), normalized, and plotted. (G-I) H460^TXR^ cells were grown for 48 h in the presence of (**G**) volasertib, (**H**) genistein, or (**I**) gefitinib at the concentration of GI_60_ of each reagent in H460^TXR^ cells. Cell lysates were subjected to a fluorometric caspase 3 activity assay. The means ± SEMs (error bars) of data from at least three experiments are shown. *, *p* < 0.05; **, *p* < 0.01; ***, *p* < 0.001 compared with control of H460 cells.

Next, to determine the effects of volasertib, genistein, and gefitinib on taxane resistance, the expression of ABC transporters was observed in NCI-H460^TXR^ cells (Figure 4A–C). *ABCB1*, a critical factor in taxane resistance, showed the highest mRNA expression among the ABC transporters in NCI-H460^TXR^ cells, which decreased markedly after treatment with volasertib, genistein, and gefitinib (Figure 4A). In addition, the levels of *ABCG2* and *ABCC9* were downregulated by treatment with these reagents compared with the control (Figure 4B,C). When the level of *PLK1* was observed to determine the effects of gefitinib and PLK1 inhibitors on PLK1 expression, downregulation was noted in NCI-H460^TXR^ cells (Figure 4D). In immunoblot analysis in NCI-H460^TXR^, the protein levels of MDR1 (encoded by ABCB1), SUR2 (encoded by ABCC9), and BCRP (encoded by ABCG2) were downregulated markedly by treatment with volasertib, genistein, and gefitinib, which was similar with those of A549^TXR^ cells (Figure 4E,F). Thus, inhibitors against PLK1 and EGFR can downregulate the expression of ABC transporters, PLK1, and EGFR, the main hurdles in paclitaxel resistance. 

Then, we wanted to investigate whether gefitinib is effective for inducing apoptotic cell death in paclitaxel-resistant lung cancer cells. For this, caspase 3 activity was measured using specific fluorogenic substrates after treatment with volasertib, genistein, and gefitinib (Figure 4G–I). The relative caspase 3 activity was higher by around 5-fold and 3-fold after treatment with volasertib in NCI-H460 cells and NCI-H460^TXR^ cells, respectively, than those of the controls (Figure 4G). After treatment with genistein, the changes in relative caspase 3 activity were similar to those produced by treatment with volasertib in NCI-H460 cells and NCI-H460^TXR^ cells (Figure 4H). The relative caspase 3 activities were higher in NCI-H460 cells than those of NCI-H460^TXR^ cells after treatment with volasertib or genistein. However, treatment with gefitinib induced apoptotic cell death more sensitively in NCI-H460^TXR^ cells than in parental cells (Figure 4I), since gefitinib treatment increased the caspase 3 activity more effectively in NCI-H460^TXR^ cells. Thus, paclitaxel-resistant lung cancers are more sensitive to gefitinib and readily undergo apoptotic cell death compared with non-resistant lung cancer, through downregulation of *ABCB1, ABCC9, ABCG2, PLK1,* and *EGFR*.

### 3.5. Clinical Relevance of EGFR and PLK1 in LUAD Cancer Patients

**Figure 5:**
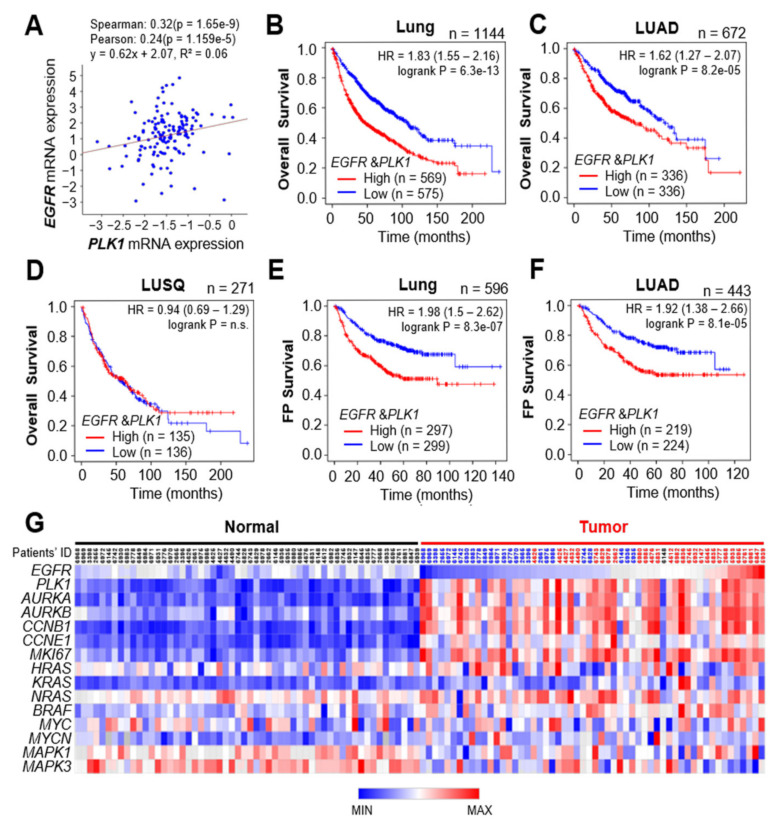
Clinical relevance of EGFR and PLK1 in LUAD cancer patients. (**A**) The relationship between the levels of *EGFR* and *PLK1* in LUAD cancer patients based on Pearson’s and Spearman’s correlation analyses from cBioPortal. (**B**–**F**) Kaplan–Meier plots representing the probability of cumulative overall survival (OS) in (**B**) lung cancer patients, (**C**) LUAD, or (**D**) LUSQ stratified according to the expression status of *PLK1* and *EGFR*. The probability of first progression (FP) survival in (**E**) lung cancer patients or (**F**) LUAD stratified according to the expression status of *PLK1* and *EGFR*. The log-rank *P*-value reflects the significance of the correlation between survival rates and the expression levels of *PLK1* and *EGFR*. HR, hazard ratio. (**G**) A heatmap was generated from Morpheus, showing the correlated expression levels of *EGFR* and *PLK1* genes in normal (Normal) and tumor (Tumor) tissues of LUAD patients. Red color of patients’ ID in tumor tissues indicates that the expression of *EGFR* is higher in tumor tissue than that in normal tissue. Blue color, lower expression; Black color, the same expression.

We further focused on the clinical relevance between expression of EGFR/PLK1 and survival rates in lung cancer patients (Figure 5). By analyzing the expression profiles of *EGFR* and *PLK1* in lung cancer patients using TCGA data from cBioPortal (Figure 5A), we found that the expression levels of *PLK1* and *EGFR* were correlated positively in lung cancer patients (Spearman factor 0.32, *p* = 1.65 × 10^−9^; Pearson factor 0.24, *p* = 1.159 × 10^−5^). In addition, for the expression levels of PLK1 and wild-type or mutant EGFR, the correlation was more significant in patients having the wild type of EGFR than in those with the mutant type (Supplementary Figure S3A–C). Based on this correlation between *PLK1* and *EGFR*, the cumulative overall survival (OS) rates were analyzed in lung cancer patients (Figure 5). The OS analysis in 1144 lung cancer patients revealed that the expression levels of *PLK1* and *EGFR* mRNA and the OS rates were correlated inversely in human lung cancer *(n* = 1144, HR = 1.83, log rank P = 6.3 × 10^−^^13^; Figure 5B). In addition, the OS rates of 672 LUAD patients were related inversely with the expression of *PLK1* and *EGFR* (*n* = 672, HR = 1.62, log rank P = 8.2 × 10^−5^), but those of 271 LUSQ patients were not (*n* = 271, HR = 0.94, log rank P = 0.72; Figure 5D). Furthermore, the survival rates until first progression (FP) showed that the expression levels of *PLK1* and *EGFR* mRNA and survival rates were correlated inversely in human lung cancer (*n* = 596, HR = 1.98, log rank P = 8.3 × 10^−7^; Figure 5E). The FP survival rates of 443 LUAD patients were related inversely with the expression of *PLK1* and *EGFR* (*n* = 443, HR = 1.92, log rank P = 8.1 × 10^−5^; Figure 5F), but those of 141 LUSQ patients were not (Supplementary Figure S3D). The expression of *PLK1* and *EGFR* was correlated inversely with the survival rates of LUAD patients. The data indicate that high expression levels of *PLK1* and *EGFR* mRNA reflect a poor prognosis in LUAD patients. Then, we sought to determine the correlation between the expression of *EGFR* and *PLK1* and tumorigenesis in lung cancer patients. For this, human LUAD data were analyzed using TCGA. The expression of *EGFR* and *PLK1* mRNA was compared in normal and tumor tissues of LUAD patients (Figure 5G). A heatmap analysis revealed that *EGFR* and *PLK1* expression was relatively higher in tumor tissues than those in normal tissues. In 29 among 56 LUAD patients, *EGFR* expression was higher in tumor tissues than in normal tissues (red color of patients’ ID in tumor tissue). In these patients, *PLK1* expression was concurrently higher in the tumor tissues than those in normal tissues. In addition, the expression of the cell proliferation markers *AURKA*, *AURKB*, *CCNB1*, and *MKI67* was higher in the patients’ tumor tissues than those of normal tissues (Figure 5G). In addition, the downstream factors of EGFR signaling including *HRAS*, *KRAS*, *NRAS*, and *BRAF* were expressed highly in LUAD patients (Figure 5G). Thus, analysis of clinical patient data provides evidence of the relevance of our findings in EGFR-positive LUAD, showing that PLK1 is a strong predictor of poor overall survival in LUAD cancer patients.

### 3.6. Combination of Gefitinib and PLK1 Inhibitor Was Effective in Paclitaxel-Resistant NCI-H460^TXR^ Cells 

**Figure 6:**
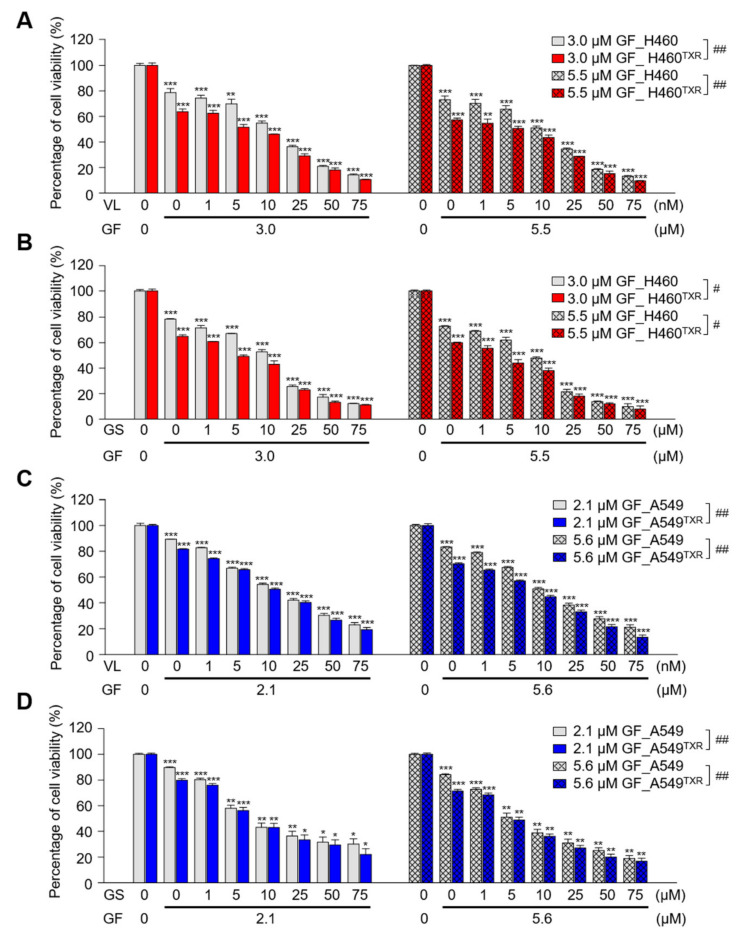
A combination of the EGFR inhibitor gefitinib and PLK1 inhibitor showed strong synergism in paclitaxel-resistant NCI-H460^TXR^ cells. (**A**,**B**) A combination between (**A**) gefitinib and (**B**) volasertib or genistein was performed in parental (H460) and paclitaxel-resistant NCI-H460 (H460^TXR^) cells in a concentration-dependent manner for 48 h. The combination of gefitinib and (**C**) volasertib or (**D**) genistein was performed in parental (A549) and paclitaxel-resistant A549 (A549^TXR^) cells in a concentration-dependent manner for 48 h. The percentages of viable cells were measured by a cell viability assay and plotted. Cells were grown for 48 h in the presence of 3.0 or 5.5 µM gefitinib with volasertib or genistein at the concentrations indicated in the figures. The percentages of viable cells were measured by cell viability assay. Three independent experiments were performed. Values are presented as means ±  SEMs (error bars). *, *p* < 0.05; **, *p* < 0.01; ***, *p* < 0.001 compared with vehicle-treated cells. #, *p* < 0.05; ##, *p* < 0.01 compared with compared with indicated groups of cells.

For a therapeutic approach to paclitaxel-resistant lung cancer, the EGFR inhibitor gefitinib and the PLK1 inhibitor volasertib or genistein were applied as a single reagent or combinatory reagents in parental and paclitaxel-resistant LUAD. First, the half maximal inhibitory concentration (IC_50_) values of gefitinib, volasertib, and genistein were evaluated in NCI-H460 and NCI-H460^TXR^ cells (Figure 6A,B; Table 1). As a single treatment, the effects of the PLK1 inhibitors volasertib and genistein were stronger in parental NCI-H460 cells than in NCI-H460^TXR^ cells, while NCI-H460^TXR^ cells were more sensitive to gefitinib treatment than were parental NCI-H460 cells (Figure 6A,B; Table 1). Then, to investigate the combined effects of gefitinib and PLK1 inhibitors, the combination index (CI) values were calculated. When the concentration of gefitinib was fixed at 3.0 μM or 5.5 μM, cell growth was inhibited by approximately 30% and 40%, respectively. Then, volasertib or genistein was administered in a concentration-dependent manner in NCI-H460 and NCI-H460^TXR^ cells (Figure 6A,B; Table 1). In NCI-H460 cells, the IC_50_ values of volasertib and genistein were 30.16 nM and 23.16 μM, respectively. These IC_50_ values were reduced to 12.79 nM and 10.78 μM, respectively, when cells were treated in combination with 3.0 μM gefitinib. The CI values between gefitinib and volasertib or genistein were 0.604 or 0.645, respectively, suggesting a synergistic effect between gefitinib and PLK1 inhibitors in NCI-H460 cells (Table 1). Moreover, these synergistic effects between gefitinib and volasertib or genistein were enhanced in paclitaxel-resistant NCI-H460^TXR^ cells. Of note, CI values between gefitinib and the PLK1 inhibitors volasertib or genistein were 0.374 and 0.355, respectively, in NCI-H460^TXR^ cells (Table 1). When volasertib and genistein were administered in combination with 5.5 μM gefitinib at GI40, the IC50 values of volasertib and genistein were reduced in NCI-H460 and NCI-H460^TXR^ cells. Under these conditions, the CI values between gefitinib and volasertib or genistein were 0.570 and 0.523, respectively (Table 1), showing more efficient synergism in paclitaxel-resistant cells compared with non-resistant cells (vs. 0.690 or 0.712), indicating that the combination effects between gefitinib and the PLK1 inhibitors were more effective in the NCI-H460^TXR^ cells. These patterns were similar in A549^TXR^ cells, which exhibited much stronger synergism between gefitinib and PLK1 inhibitors, with CI values of approximately 0.2 (vs. the CIs of 0.5~0.7 in A549 cells; Figure 6C,D; Table 2). In summary, the combination of gefitinib and PLK1 inhibitor can increase the therapeutic sensitivity of LUAD^TXR^ cells showing strong synergism. 

### 3.7. Inhibition of EGFR and PLK1 Effectively Reduced the Expression of ABC Transporters by Suppressing MYC and FOS/JUN in Paclitaxel-Resistant LUAD Cells

**Figure 7:**
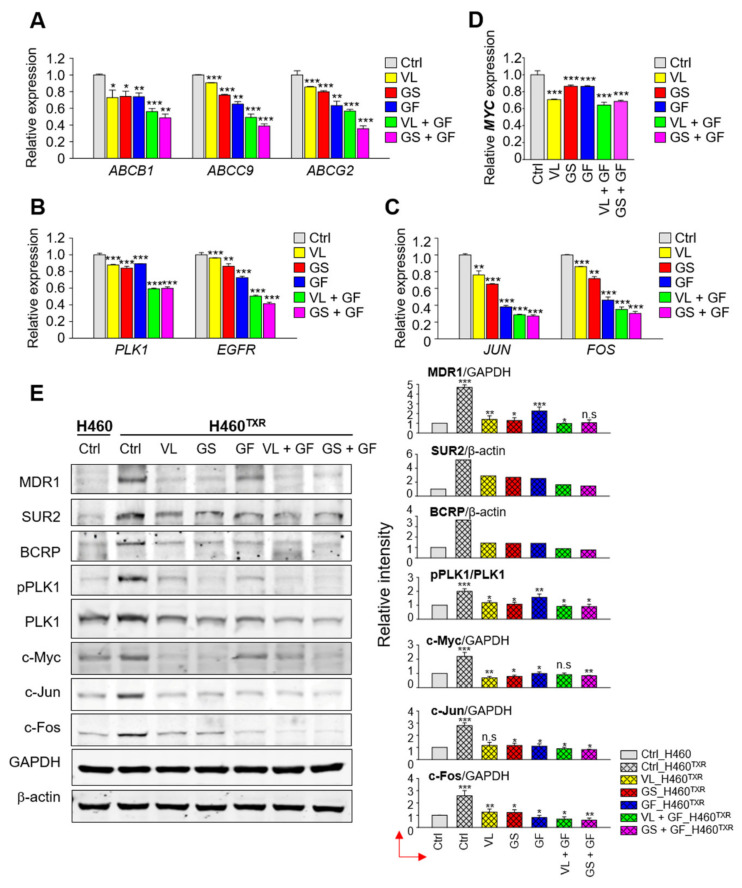
A combination of gefitinib and PLK1 inhibitors effectively reduced the expression of ABC transporters by suppressing *EGFR*, *PLK1*, and *MYC* in paclitaxel-resistant NCI-H460^TXR^ cells. Paclitaxel-resistant NCI-H460 (H460^TXR^) cells were grown for 48 h in the presence of 51.4 nM volasertib, 37.8 µM genistein, or 12.1 µM gefitinib at the concentration of GI_50_ for the single treatments. For the combination treatment at the concentration of GI_50_ in TXR cells, 8.1 nM volasertib or 6.9 µM genistein was co-administered with 1.5 µM gefitinib (GI_20_). Quantitative RT-PCR was performed to evaluate the mRNA levels of (**A**) *ABCB1, ABCC9,* and *ABCG2*, (**B**) *PLK1* and *EGFR*, (**C**) *JUN* and *FOS*, and (**D**) *MYC*. The relative expression levels of mRNA were plotted. Three independent experiments were performed. *, *p* < 0.05; **, *p* < 0.01; ***, *p* < 0.001. (**E**) Cell lysates were subjected to immunoblotting with anti-MDR1, anti-SUR2, anti-BCRP, anti-PLK1, anti-p-PLK1, anti-c-Myc, anti-c-Jun, anti-c-Fos, anti-GAPDH, and anti-β-actin antibodies. The relative intensities were quantified using LI-COR Odyssey software (Li-COR Biosciences), normalized, and plotted. *, *p* < 0.05; **, *p* < 0.01; ***, *p* < 0.001 compared with control of cells.

To further investigate whether the combination of gefitinib and PLK1 inhibitor can downregulate the expression of ABC transporters, qRT-PCR was performed at half maximal cell growth in both single and combination treatments. For the single treatment at the concentration of GI_50_, NCI-H460^TXR^ cells were grown for 48 h in the presence of 51.39 nM volasertib, 37.84 µM genistein, and 12.06 µM gefitinib (Table 3; Supplementary Figure S4). For the combination that induced half maximal cell growth in NCI-H460^TXR^ cells, 1.5 µM gefitinib was co-administered with 8.13 nM volasertib or 6.86 µM genistein (Table 3; Supplementary Figure S4). Although cell death effects were similar at 50% for single or combination treatment, the expression of *ABCB1, ABCC9*, or *ABCG2* in the combination treatment was reduced markedly compared with those of single treatment in qRT-PCR analysis (Figure 7A). In addition, the expression of *PLK1* and *EGFR* was downregulated effectively by a combination treatment (Figure 7B). The patterns of downregulation were similar with those of A549^TXR^ cells (Supplementary Figure S5A,B). The downregulation patterns of *EGFR* were similar to those of *ABCG2* and *ABCC9* after treatment with inhibitors, while those of *PLK1* were similar to those of *ABCB1*. 

Emerging evidence indicates that ABC transporters can be regulated transcriptionally by AP-1, a transcriptional activating complex of c-Jun/c-Fos [30,31,32,33], or c-Myc [34,35,36]. In addition, EGFR tyrosine kinase and autophosphorylation at Y1173 are crucial for activation of AP-1 [37]. To further investigate how ABC transporters can be suppressed effectively by the combination of gefitinib and PLK1 inhibitor in LUAD^TXR^ (Figure 7, Supplementary Figure S5), the transcriptional factors AP-1 and c-Myc were observed. The levels of *FOS* and *JUN* were downregulated similarly to those of *EGFR, ABCG2*, and *ABCC9* by treatment with volasertib, genistein, and gefitinib as single or combination treatments (Figure 7C, Supplementary Figure S5C), suggesting that activation of AP-1 can affect the levels of *ABCG2* and *ABCC9*. In addition, the level of *MYC* was downregulated similarly to those of *PLK1* and *ABCB1* (Figure 7D, Supplementary Figure S5D). Since the stability of Myc was regulated directly or indirectly by PLK1 for tumor survival [34,38,39,40], the protein level of c-Myc was observed by immunoblot analysis (Figure 7E, Supplementary Figure S5E). In the immunoblot, the level of c-Myc was downregulated markedly by treatment with PLK1 inhibitors but not by gefitinib, which was similar to the response of MDR1 (ABCB1), SUR2 (ABCC9), and BCRP (ABCG2) (Figure 7E, Supplementary Figure S5E). In addition, the level of p-T210-PLK1, the active form of PLK1, was similar to that of c-Myc after treatment with PLK1 inhibitors as a single or combination treatment. Therefore, PLK1 inhibitors downregulated the levels of p-PLK1 and c-Myc, which reduced the expression of MDR1 in LUAD^TXR^. However, c-Jun and c-Fos levels were reduced markedly even in cells treated solely with gefitinib, similar to the mRNA alterations of qRT-PCR (Figure 7C,E, Supplementary Figure S5), indicating that EGFR inhibition suppressed AP-1 and, consequently, ABC transporters including *ABCG2* and *ABCC9* of its transcriptional targets. In other words, AP-1 regulated the expression of *ABCC9* and *ABCG2* in LUAD^TXR^. From the data, in taxane-related chemoresistant LUAD, the PLK1/c-Myc axis can regulate the expression of *ABCB1*, and the EGFR/AP-1 axis can affect the expression of *ABCG2* and *ABCC9*.

Taken together, these results show that a combination of gefitinib and the PLK1 inhibitor volasertib or genistein exerts strong synergism in LUAD^TXR^ by suppressing the activities of EGFR, PLK1, c-Myc, or AP-1, leading to downregulation of ABC transporters.

## 4. Discussion

Previously, we found that paclitaxel resistance resulted in high expression of PLK1, a mitotic master kinase for cell division [17,27]. Paclitaxel, a microtubule-stabilizing agent, leads to mitotic arrest and upregulation of PLK1 due to interference with cell division in cancer cells [17,25,26]. When PLK1 was blocked using specific inhibitors, the expression of ABC transporters and cell viability were downregulated effectively [17,27]. Based on previous studies, we hypothesized that active PLK1 can upregulate the expression of ABC transporters, which can be downregulated by PLK1 inhibition. In this study, we demonstrated that the activity or presence of PLK1 is correlated directly with the expression of ABC transporters. To explore this, various versions of PLK1 including a constitutively active mutant, kinase-dead mutant, and substrate-binding domain mutant were expressed in LUAD^TXR^, and their effects on the expression of ABC transporters were observed. The expression of active PLK1 upregulated the levels of ABC transporters, which were downregulated by non-functional PLK1 in LUAD^TXR^. The downregulation effects for ABC transporters were correlated positively with the sensitivity for apoptotic cell death in LUAD^TXR^. In addition, depletion of PLK1 using shRNA reduced the expression of ABC transporters in LUAD^TXR^. From these findings, we suggest that the activity and expression of PLK1 are crucial to regulate the expression of ABC transporters to increase chemoresistance in LUAD^TXR^.

To explore the exact regulatory mechanism for the expression of *ABCB1*, a main transporter, in paclitaxel-resistant LUAD by active PLK1, downregulation of c-Myc has been observed after treatment with a PLK1 inhibitor. c-Myc has been explored as a main transcriptional factor for stemness and *ABCB1* expression [34,35,36,41], while PLK1 is essential for stabilization of c-Myc for tumor survival by phosphorylation-mediated stabilization [38,39,40]. PLK1 promotes Fbw7 phosphorylation, self-ubiquitination, and proteasomal degradation, creating a PLK1-Myc feedforward activation loop in *MYC* overexpressing tumor cells [38]. In addition, emerging evidence suggests that the Fbw7-PLK1 axis regulates the response to paclitaxel because loss of Fbw7 and accumulation of PLK1 promote paclitaxel resistance in breast cancer [42]. Based on the evidence, PLK1 can stabilize c-Myc through phosphorylation of Fbw7 for its dissociation with c-Myc, which translocalizes into the nucleus and upregulates the expression of ABC transporters. In the study, we revealed that inhibition of PLK1 reduced the level of c-Myc, leading to suppression of *ABCB1*. This is plausible as one of the regulatory mechanisms for how PLK1 upregulates ABC transporter expression. Here, PLK1 inhibition downregulated the level of *MYC*, which reduced the expression of ABC transporters, especially that of *ABCB1* (Figure 7). However, EGFR inhibition using gefitinib did not markedly reduce the levels of c-Myc and MDR1. These data suggest that the expression of *ABCB1* is regulated by the axis of PLK1-MYC in chemoresistant LUAD. 

**Figure 8:**
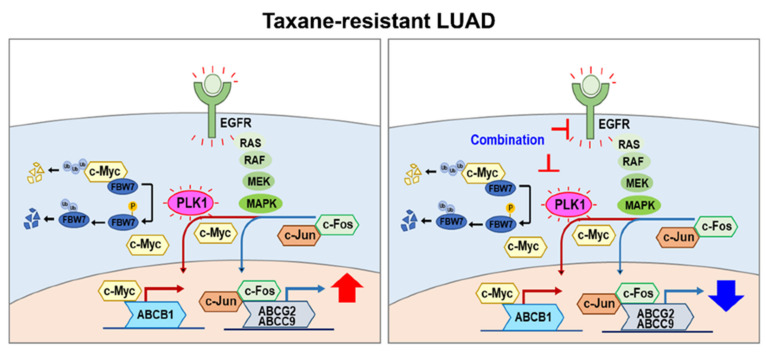
Plausible model of a combination effect using gefitinib and PLK1 inhibitor in taxane-resistant LUAD cells.

Although the expression of *ABCB1* is regulated by the PLK1 and c-Myc signaling axis, expression of *ABCC9* and *ABCG2* was not altered dramatically by PLK1 inhibition. Instead, their expression levels were sensitive to the EGFR inhibitor gefitinib. The expression patterns of *EGFR*, *ABCC9*, and *ABCG2* were similar when inhibitors for EGFR and PLK1 were administered as a single or combination treatment (Figure 7). Previous studies reported that AP-1, a transcriptional activating complex of c-Jun/c-Fos, is a regulator for the expression of ABC transporters including *ABCG2* [30,31,32,33]. *ABCG2* contains AP-1 binding sites at the promoter region located approximately 312 bp from the transcriptional start site [32]. In addition, the promoter of *ABCC9* is activated via AP-1 signaling in hypoxia H9c2 cells [33]. Moreover, EGFR tyrosine kinase and autophosphorylation at Y 1173 are crucial for activation of AP-1 [37]. In this study, we demonstrated that targeting EGFR using gefitinib is important to downregulate the expression of FOS (encoding c-Fos, a component of AP-1) to reduce the levels of *ABCG2* and *ABCC9* (see Figure 8). Therefore, activation of EGFR is a crucial factor for expression of ABC transporters including *ABCG2* through AP-1 activation. 

As a molecular marker of LUAD, the expression levels of EGFR and its mutation are targetable indices to determine first-line treatment of LUAD [4]. A recent study showed that siPLK1 and EGFR-targeted nanoparticles improved radiation sensitivity in NSCLC [43]. In addition, PLK1 inhibition can reduce EGFR resistance due to its inhibitors or mutation at T790M in NSCLC [44]. These previous studies support PLK1 and EGFR co-targeting as a valuable approach for advanced NSCLC. As shown in the clinical relevance part of this study, TCGA analysis revealed that EGFR and PLK1 expression was relatively higher in the tumor tissues of LUAD patients compared with normal tissues. In addition, the KM plot analysis of patient data demonstrates PLK1 as a strong predictor of poor overall survival in EGFR-positive LUAD patients. Because EGFR expression was much higher in LUAD^TXR^ compared with non-resistant LUAD, the EGFR-specific inhibitor gefitinib was administered, resulting in downregulation of EGFR in LUAD^TXR^ more sensitively than that in non-resistant LUAD. The upregulated ABC transporters also were downregulated by administration of PLK1 inhibitors or gefitinib, suggesting that EGFR and PLK1 inhibitors reduced paclitaxel resistance by reducing the expression levels of ABC transporters. 

Combination treatment with PLK1 inhibitors and gefitinib increased the therapeutic sensitivity of paclitaxel-resistant LUAD by decreasing ABC transporter expression. Analysis of clinical patient data provided evidence for the particular relevance of our findings in LUAD having the wild type of EGFR, showing that PLK1 is a valuable target for reducing chemoresistance. Therefore, dual inhibition of PLK1 and EGFR can show a highly synergistic effect by reducing the ABC transporters through downregulation of c-Myc or AP-1 in taxane-resistant LUAD.

## 5. Conclusions

In this study, we found that the activity and expression of PLK1 are crucial factors for the expression of ABC transporters in LUAD. Since abnormal activation of EGFR is an important element to determine the first line of chemotherapy, co-targeting EGFR and PLK1 is a valuable strategy for the treatment of LUAD. Notably, the combination of PLK1 inhibitor and gefitinib potentiates the therapeutic sensitivity of paclitaxel-resistant LUAD by decreasing ABC transporter expression through c-Myc or AP-1 signaling. Therefore, the dual targeting of PLK1 and EGFR exerts strong synergism in LUAD^TXR^, which provides a benefit to overcome the limitations associated with taxanes. Further in clinical studies are needed to validate the therapeutic synergism in taxane-resistant LUAD.

## Figures and Tables

**Table 1:** The half maximal inhibitory concentration (IC_50_) values of gefitinib, volasertib, and genistein and the combination index (CI) values in NCI-H460 and NCI-H460^TXR^ cells.

Compounds	NCI-H460		NCI-H460^TXR^	
	IC_50_	CI	IC_50_	CI
Gefitinib (µM)	16.68	-	12.06	-
Volasertib (nM)	30.16	-	51.39	-
Volasertib (in combination 3.0 µM Gefitinib)	12.79	0.604	6.41	0.374
Volasertib (in combination 5.5 µM Gefitinib)	10.85	0.690	5.85	0.570
Genistein (µM)	23.16	-	37.84	-
Genistein (in combination 3.0 µM Gefitinib)	10.78	0.645	4.02	0.355
Genistein (in combination 5.5 µM Gefitinib)	8.85	0.712	2.54	0.523

**Table 2:** The half maximal inhibitory concentration (IC_50_) values of gefitinib, volasertib, and genistein and the combination index (CI) values in A549 and A549^TXR^ cells.

Compounds	A549		A549^TXR^	
	IC_50_	CI	IC_50_	CI
Gefitinib (µM)	19.91	-	43.17	-
Volasertib (nM)	21.63	-	67.27	-
Volasertib (in combination 2.1 µM Gefitinib)	14.16	0.760	10.42	0.204
Volasertib (in combination 5.6 µM Gefitinib)	10.33	0.759	7.48	0.241
Genistein (µM)	17.10	-	54.42	-
Genistein (in combination 2.1 µM Gefitinib)	7.16	0.524	6.71	0.172
Genistein (in combination 5.6 µM Gefitinib)	4.92	0.569	4.09	0.205

**Table 3:** The half maximal inhibitory concentration (IC_50_) values of gefitinib, volasertib, and genistein and the combination index (CI) values when 1.5 µM of gefitinib (GI_20_) was combined with volasertib and genistein in NCI-H460^TXR^ cells.

Compounds	NCI-H460^TXR^	
	IC_50_	CI
Gefitinib (µM)	12.06	-
Volasertib (nM)	51.39	-
Genistein (µM)	37.84	-
Volasertib (in combination 1.5 µM Gefitinib)	8.13	0.283
Genistein (in combination 1.5 µM Gefitinib)	6.86	0.306

## Supplementary Materials

The following are available online at https://www.mdpi.com/article/10.3390/cancers13174413/s1, Figure S1: The half maximal inhibitory concentration (IC_50_) of paclitaxel was higher in paclitaxel-resistant NCI-H460 cells expressing active PLK1 than it was in cells expressing non-functional PLK1, Figure S2: The half maximal inhibitory concentration (IC_50_) values of PLK1 inhibitors were higher in paclitaxel-resistant NCI-H460 cells than in parental cells, Figure S3: Clinical relevance of the levels of *EGFR* and *PLK1* in lung cancer patients, Figure S4: A combination of EGFR inhibitor gefitinib and PLK1 inhibitor in paclitaxel-resistant NCI-H460^TXR^ cells, Figure S5: A combination of gefitinib and PLK1 inhibitors effectively reduced the expression of ABC transporters by suppressing *EGFR*, *PLK1*, and *MYC* in paclitaxel-resistant A549^TXR^ cells, Table S1: Sequences of forward and reverse primers used for qRT-PCR amplification.
