# Supplementary material for: Dual Targeting of EGFR with PLK1 Exerts Therapeutic Synergism in Taxane-Resistant Lung Adenocarcinoma by Suppressing ABC Transporters
Source: Cancers (Basel). 2021 Sep 1;13(17):4413. doi: 10.3390/cancers13174413 (PMC8430738; doi:10.3390/cancers13174413)
Supplement: Supplementary file 1 [file cancers-13-04413-s001.zip › cancers-1279215-SI.pdf]

Supplementary Information

Dual Targeting of EGFR with PLK1 Exerts Therapeutic Synergism in Taxane-Resistant Lung Adenocarcinoma by Suppressing ABC Transporters

Sol-Bi Shin [1,†], Dae-Hoon Kim [1,†], Da-Eun Kim [1], Mark Borris D. Aldonza [2], Yoosik Kim [2] and Hyungshin Yim [1,*]

[1] Department of Pharmacy, College of Pharmacy, Institute of Pharmaceutical Science and Technology, Hanyang University, Ansan, Gyeonggi-do 15588, Korea; solbi@hanyang.ac.kr (S.-B.S.); kdhsh100@hanyang.ac.kr (D.-H.K.); daeunyh@hanyang.ac.kr (D.-E.K.)

[2] Department of Chemical and Biomolecular Engineering, Korea Advanced Institute of Science and Technology (KAIST), Daejeon 34141, Korea; borris@alumni.kaist.ac.kr (M.B.D.A.); ysyoosik@kaist.ac.kr (Y.K.)

[†] These authors contributed equally to this work.

[*] Correspondence: hsyim@hanyang.ac.kr; Tel.: +82-31-400-5810

Supplementary Figures and Table

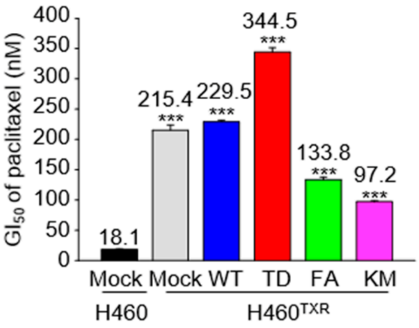

Supplementary Figure S1. The half maximal inhibitory concentration (IC_{50}) of paclitaxel was higher in paclitaxel-resistant NCI-H460 cells expressing active PLK1 than it was in cells expressing non-functional PLK1. ERFP-tagged vector (Mock), wild-type (WT), polo-box mutant (FA), or kinase-dead K82M (KM) variant of PLK1 was expressed in parental or paclitaxel-resistant NCI-H460 ($H460^{TXR}$) cells. Cells were selected with puromycin. NCI-H460 and $H460^{TXR}$ cells were grown for 48 hours in the presence of 5, 10, 25, 50, 100, 250, or 500 nM paclitaxel. The percentages of viable cells were measured by cell viability assay, and the values of GI_{50} were plotted. Three independent experiments were performed. ***, p<0.001.

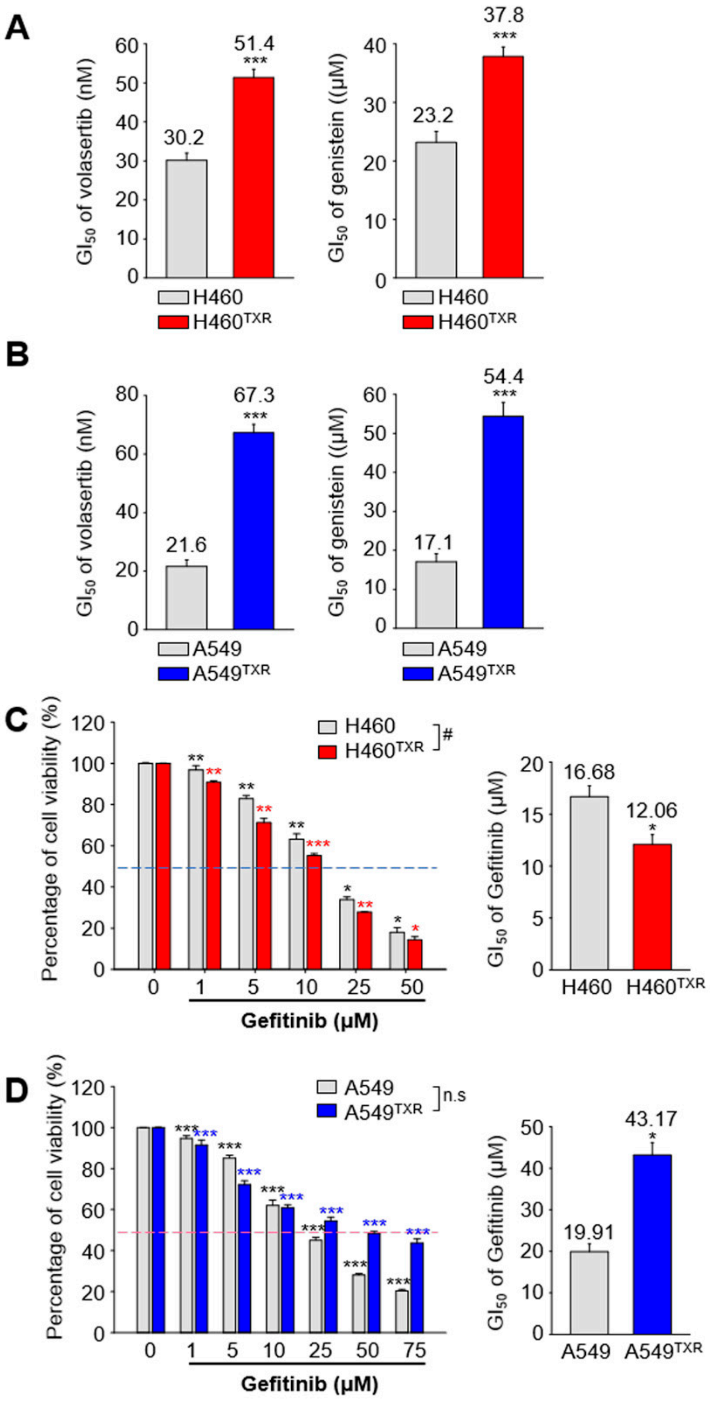

Supplementary Figure S2. **The half maximal inhibitory concentration (IC_{50}) values of PLK1 inhibitors were higher in paclitaxel-resistant NCI-H460 cells than in parental cells.** **(A)** NCI-H460 and NCI-H460^{TXR} cells and **(B)** A549 and A549 ^{TXR} cells were grown for 48 hours in the presence of 1, 5, 10, 25, 50, or 75 nM volasertib or 1, 5, 10, 25, 50, or 75 µM genistein for 48 hours**.** The percentages of viable cells were measured by cell viability assay, and the values of GI_{50} were plotted. Three independent experiments were performed. ***, $p<0.001$. **(C)** NCI-H460 and NCI-H460^{TXR} cells and **(D)** A549 and A549 ^{TXR} cells were grown for 48 hours in the presence of 1, 5, 10, 25, and 50 µM gefitinib. The bar graph presents the mean values of half maximal growth inhibitory concentration (GI_{50}, µM). *, $p<0.05$; **, $p<0.01$; ***, $p<0.001$ compared with control (vehicle-treated). #, $p<0.05$; ##, $p<0.01$ compared with mock of parental H460 or A549 cells.

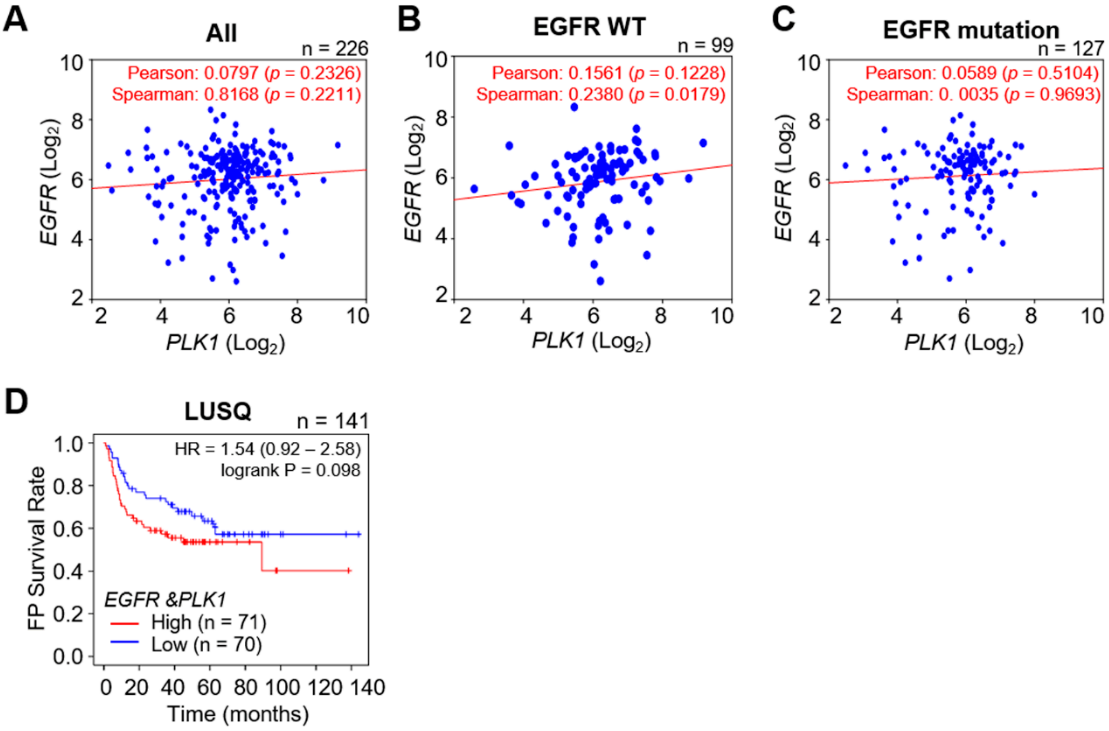

Supplementary Figure S3. **Clinical relevance of the levels of *EGFR* and *PLK1* in lung cancer patients.** The correlation between the levels of *EGFR* and *PLK1* in **(A)** lung cancer patients (All), **(B)** patients having wild-type EGFR, or **(C)** patients having a mutant of EGFR according to Pearson and Spearman's correlation analyses from the R statistical software using TCGA patient data. **(D)** Kaplan–Meier plots representing the probability of first progression (FP) survival in patients with LUSQ stratified according to the expression status of *PLK1* and *EGFR*. Log-rank *P*-values reflect the significance of the correlation between survival rates and the expression of *PLK1* and *EGFR*. HR, hazard ratio

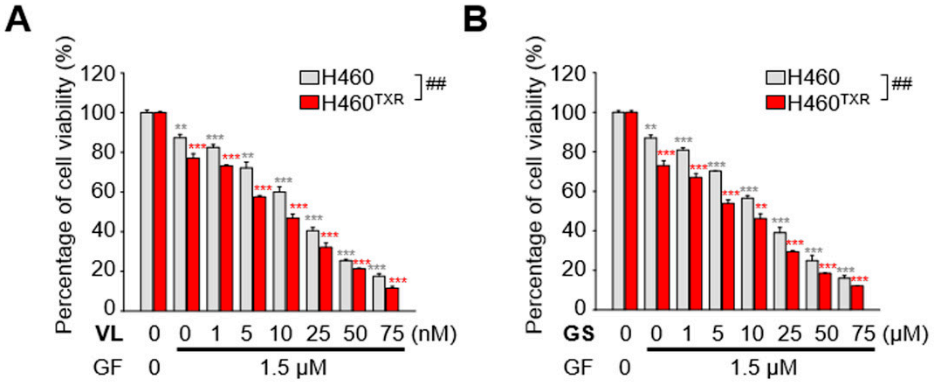

Supplementary Figure S4. A combination of EGFR inhibitor gefitinib and PLK1 inhibitor in paclitaxel-resistant NCI-H460^{TXR} cells. A combination of gefitinib and **(A)** volasertib or **(B)** genistein was administered to parental (H460) and paclitaxel-resistant NCI-H460 (H460^{TXR}) cells in a concentration-dependent manner for 48 hours. Cells were grown for 48 hours in the presence of 1.5 µM gefitinib (GI_{20}) with volasertib or genistein at the indicated concentrations. The percentages of viable cells were measured by cell viability assay. Three independent experiments were performed. Values are presented as mean + standard deviation. *, p<0.05; **, p<0.01; ***, p<0.001 compared with control of each cells. #, p<0.05; ##, p<0.01 compared with mock of parental H460.

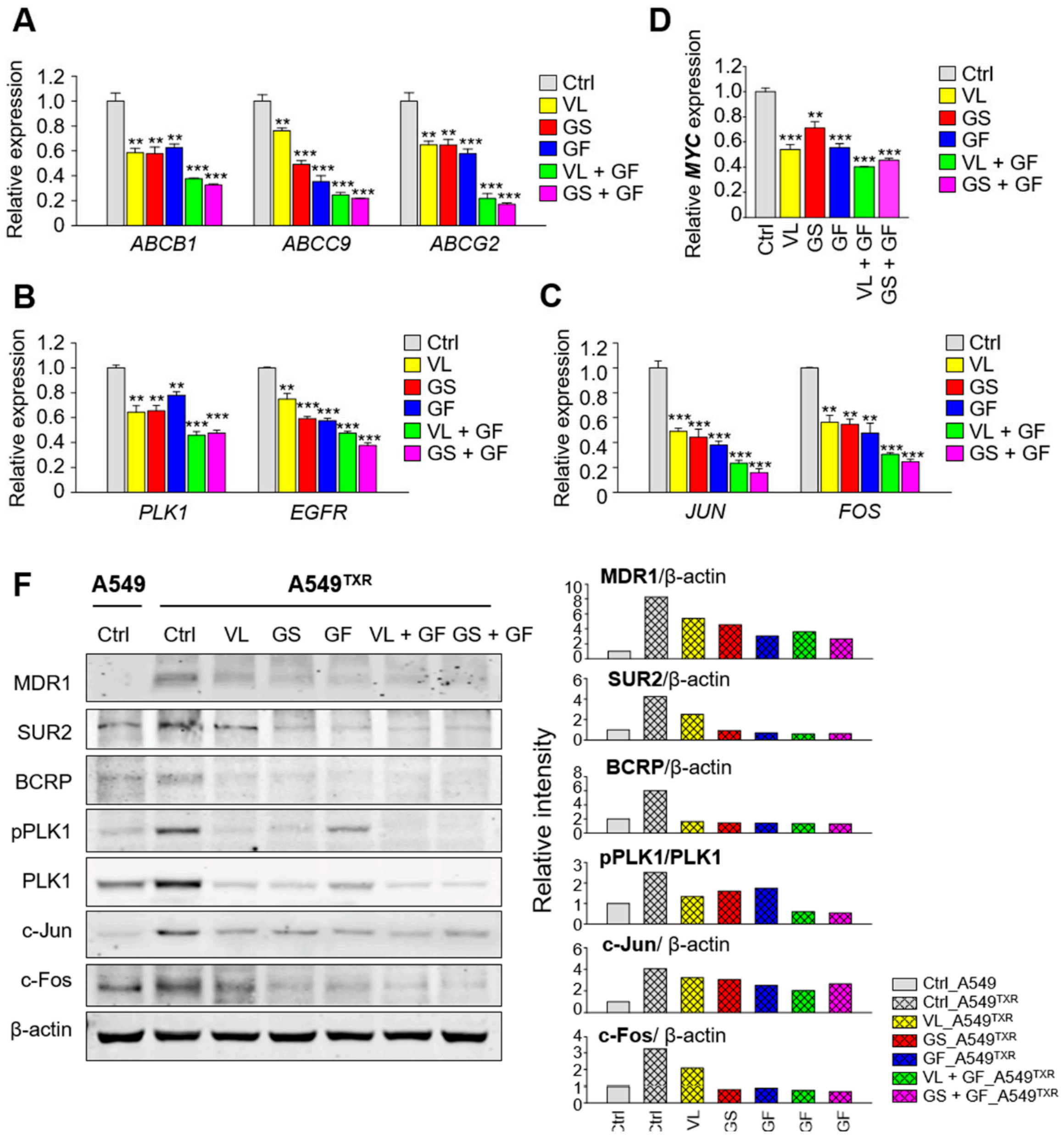

Supplementary Figure S5. A combination of gefitinib and PLK1 inhibitors effectively reduced the expression of ABC transporters by suppressing *EGFR*, *PLK1*, and *MYC* in paclitaxel-resistant A549^{TXR} cells. Paclitaxel-resistant A549 (A549^{TXR}) cells were grown for 48 hours in the presence of 64.7 nM volasertib, 61.6 µM genistein, or 38.1 µM gefitinib at the concentration of GI_{50} for the single treatments. For the combination treatment at the concentration of GI_{50} in TXR cells, 14.2 nM volasertib or 7.2 µM genistein was co-administered with 2.1 µM gefitinib (GI_{20}). Quantitative RT-PCR was performed to evaluate the mRNA levels of **(A)** *ABCB1, ABCC9,* and *ABCG2*, **(B)** *PLK1* and *EGFR*, **(C)** *JUN* and *FOS*, and **(D)** *MYC*. The relative expression levels of mRNA were plotted. Three independent experiments were performed. *, $p<0.05$; **, $p<0.01$; ***, $p<0.001$. **(E)** Cell lysates were subjected to immunoblotting with anti-MDR1, anti-SUR2, anti-BCRP, anti-PLK1, anti-p-PLK1, anti-c-Jun, anti-c-Fos, and anti-β-actin antibodies. The relative intensities were quantified using LI-COR Odyssey software (Li-COR Biosciences), normalized, and plotted. *, $p<0.05$; **, $p<0.01$; ***, $p<0.001$ compared with control of cells.

Supplementary Table S1. Sequences of forward and reverse primers used for qRT-PCR amplification.

**Target Gene**	**Primer**	**Sequences**
Human *PLK1*	Forward<br>Reverse	5’- AAGAGATCCCGGAGGTCCTA -3’<br>5’- TCATTCAGGAAAAGGTTGCC -3’
Human *ABCB1*	Forward<br>Reverse	5’- ATATCAGCAGCCCACATCAT -3’<br>5’- GAAGCACTGGGATGTCCGGT -3’
Human *ABCC9*	Forward<br>Reverse	5’-CCTCTTTATGCCAGCCGTGA -3’<br>5’- CTGTGATGCAGAAACGCAGG -3’
Human *ABCG2*	Forward<br>Reverse	5’- TGGAATCCAGAACAGAGCTGG -3’<br>5’- AGTGTTTCAGCCGTGGAACTCT -3’
Human *EGFR*	Forward<br>Reverse	5’ -CGTTCGGCACGGTGTATAA -3’<br>5’- GGCTTTCGGAGATGTTGCTTC -3’
Human *GAPDH*	Forward<br>Reverse	5’- TAAAGGGCATCCTGGGCTACACT -3’<br>5’- TTACTCCTTGGAGGCCATGTAGG -3’
Human *MYC*	Forward<br>Reverse	5’- AAACACAAACTTGAACAGCTAC -3’<br>5’- ATTTGAGGCAGTTTACATTATGG -3’
